# Towards the multileveled and processual conceptualisation of racialised individuals in biomedical research

**DOI:** 10.1007/s11229-022-04004-2

**Published:** 2022-12-28

**Authors:** Joanna Karolina Malinowska, Tomasz Żuradzki

**Affiliations:** 1 Faculty of Philosophy, Adam Mickiewicz University, ul. Szamarzewskiego 89C, 60-568 Poznań, Poland; 2 Institute of Philosophy & Interdisciplinary Centre for Ethics, Jagiellonian University, ul. Grodzka 52, 31-044 Kraków, Poland

**Keywords:** Biomedical research, Race, Ethnicity, Racialisation, Racialised individuals

## Abstract

In this paper, we discuss the processes of racialisation on the example of biomedical research. We argue that applying the concept of racialisation in biomedical research can be much more precise, informative and suitable than currently used categories, such as race and ethnicity. For this purpose, we construct a model of the different processes affecting and co-shaping the racialisation of an individual, and consider these in relation to biomedical research, particularly to studies on hypertension. We finish with a discussion on the potential application of our proposition to institutional guidelines on the use of racial categories in biomedical research.

## Introduction

Although race and ethnicity are rather controversial, raw and imprecise proxies, they have had an influence on biomedical research and medical practice for years. They are regularly accepted in epidemiology and public health surveillance, especially in the United States (US).^1^ They are commonly applied as a reference class in randomised control trials (RCTs), polygenic risk scores (PRS) or diagnostic algorithms. Since 2001, the National Institutes of Health (NIH) has required the use of these categories to collect and report data in submissions for clinical trials.^2^ Similarly, since 2005, the Food and Drug Administration (FDA) in the US has recommended the collection and use of racial and ethnic data (both institutions, the NIH and the FDA, follow the typology endorsed by the Office of Management and Budget).^3^ In the recent ‘Guidance for Industry and Food and Drug Administration Staff’ on *Collection of Race and Ethnicity Data in Clinical Trials* (FDA, 2016),^4^ it is recommended that researchers categorise study participants into at least five racial (American Indian or Alaska Native, Asian, Black or African American, Native Hawaiian or Other Pacific Islander and White) and two ethnic (Hispanic or Latino and Not Hispanic or Latino) categories.

Although the use of ethnoracial categories^5^in medicine has been prevalent for years, no standard definition of race has been developed, and thus this term is interpreted and documented inconsistently in different studies and legal frameworks (Baer et al., 2013; López et al., 2017; Malinowska & Żuradzki, 2022; Popejoy et al., 2020; Singh & Steeves, 2020; Zhang & Finkelstein, 2019). In some cases, it is naturalised (e.g. biologised), and at other times it is considered a purely culturally based concept. In the FDA’s, 2016 guidelines, race is interpreted as a historico-geographical concept referring to a subpopulation whose representatives have common ancestors inhabiting specific geographical areas.^6^ This thesis can be seen in the text of the FDA guidelines, where examples of racial differences in responses to medical treatments are listed. An FDA review of drug approvals between 2008 and 2013 found that approximately one-fifth of new drugs demonstrated some differences in exposure and/or response across racial/ethnic groups (Ramamoorthy et al., 2015). For example, racial differences in skin structure and physiology can affect response to dermatological and topically applied products (Taylor, 2002). The mortality rates of patients on dialysis have been shown to differ across race and ethnicity groups:

Collecting data on race and/or ethnicity is critical to identifying population-specific signals. As illustrated above, genetic studies may explain the basis for observed differences in pharmacokinetics, efficacy, or safety across racial or ethnic subgroups, and FDA has recommended collection of DNA samples in clinical trials for such purposes. FDA has also published guidance on enrichment strategies for selecting participants for clinical trials based on prognostic or predicative biomarkers (such as genotype). (FDA, 2016, pp. 7–8)

Although the FDA seems to naturalise race, in the same document the agency admits that these differences may be attributable to different internal (e.g. genetic, metabolic, ‘elimination’) and external factors (e.g. diet, environmental exposure, socio-cultural), as well as interactions between them (FDA, 2016, p. 7), with the main emphasis on genetics. This approach exemplifies the thesis that the category of race is commonly used as a population descriptor in the study of genomic variation or ‘as surrogates for ancestral background’ (Bonham et al., 2018).

The use of racial classifications in research is also co-shaped by the editorial policies of scientific journals. For example, the *Journal of the American Medical Association* published their ‘Updated Guidance on the Reporting of Race and Ethnicity in Medical and Science Journals’ (Flanagin et al., 2021a; cf. Flanagin et al., 2021b). The definitions of race presented in this information are (compared with the FDA’s guidelines) much more constructivist:

1. Race and ethnicity are social constructs, without scientific or biological meaning (Flanagin et al., 2021a, p. 621).
2. Race and ethnicity are dynamic, shaped by geographic, cultural, and socio-political forces. Race and ethnicity are social constructs and with limited utility in understanding medical research, practice, and policy. However, the terms may be useful as a lens through which to study and view racism and disparities and inequities in health, health care, and medical practice, education, and research (Flanagin et al., 2021a, p. 622).

This approach corresponds better with the original NIH policy document from 2001, which states that racial and ethnic ‘categories in this classification are social-political constructs and should not be interpreted as anthropological in nature’.^7^ The authors of the JAMA guidance state outright that the inclusion of race and ethnicity in medical research reports is supposed ‘to address and further elucidate health disparities and inequities [and] remains important at this time’ (Flanagin et al., 2021a, p. 622), rather than grabbing any innate biological features of the representatives of different populations. They also note that the terms ‘ancestry’ (defined as ‘a person’s country or region of origin or an individual’s lineage of descent’) and ‘genetic admixture’ (defined as ‘genetic exchange among people from different ancestries’) may provide much more useful and reliable information about ‘health, population health, and genetic variants and risk for disease or disorders than do racial and ethnic categories’ (Flanagin et al., 2021a, p. 622). However, it is worth considering whether, if the categories of ‘ancestry’ and ‘genetic admixture’ are indeed more precise than ‘race’ regarding genetic and genomic research, ‘race and ethnicity’ can still be used within these fields. It seems that the authors of the guidelines give a positive answer to this question. Flanagin et al. (2021a) discuss racial categories in the context of people’s geographic origins as well as genetic and genomic research. As an example of the correct reporting of the results of research relating to racial categories, they quote from a study conducted by Machipisa et al. (2021):

In this genome-wide association study, participants were from eight African countries (i.e., Kenya, Mozambique, Namibia, Nigeria, South Africa, Sudan, Uganda, and Zambia). Any Black African group from any of the eight African countries (mostly of Bantu descent) was included in the Black African cohort. The South African group composed primarily of multiple racial categories, comprising any admixture combination of individuals of European, Southeast Asian, South Asian, Bantu-speaking African, and/or indigenous Southern African hunter-gatherer ancestries (Khoikhoi, San, or Bushmen), was renamed admixed African individuals. The race and ethnicity of an individual was self-reported.

It is understandable that in case of such diverse reference classes, it should be emphasised that they comprise an admixture combination of individuals from different ancestries. However, the very use of the terms ‘race and ethnicity’ and ‘racial categories’ (previously defined as social constructs) in the case of genomic study raises concerns, as it gives them a biological overtone.

The authors of the guidelines also note the difficulties involved in analysing the complex impact of ‘race’ on human health:

There are many examples of reported associations between race and ethnicity and health outcomes, but these outcomes may also be intertwined with ancestry and heritage, social determinants of health, as well as socioeconomic, structural, institutional, cultural, demographic, or other factors. Thus, discerning the roles of these factors is difficult. (Flanagin et al., 2021a, p. 622)

Grasping the nature of the associations between a person’s racial affiliation and their health is, in fact, a great challenge. The analysis of such a multitude of factors (socio-cultural and biological) and the interactions influencing human health considered through the prism of racial classifications (or with the associations with them) lead to many difficulties and much confusion. For example, it is frequently hard to infer what the term ‘race’ refers to when reading one of the many scientific articles containing the phrase ‘racial differences’ in the title.^8^ Because of this it may also be difficult to understand what causes these ‘racial differences’ and whether researchers analyse this problem from a genetic, cultural or environmental angle, or maybe from a viewpoint combining one or more of these perspectives. We want to contribute to solving this problem.

In our paper, we develop the concept of racialisation and discuss its application in biomedical research in the example of studies on hypertension. Racialisation is a process by which groups of individuals are understood by others, as well as recognised by themselves, as representatives of major biological entities and human lineages, usually called races (Hochman, 2017; Flanagin et al., 2021a, 2021b). The concept of racialisation can be used, for example, to analyse the impact of systemic racism on health without reifying the category of race.

Our work is supposed to contribute to the task of sorting out the inaccuracies and imprecision in the conceptual apparatus used in biomedical science (thus, our task is both descriptive and normative/ameliorative). It fits well within the framework of conceptual engineering (Anderson et al., 2012; Burgess et al. 2020; Eklund, 2021; Haslanger, 2000, 2008, 2010; Oswick et al., 2021), the aims of which are: ‘(i) The assessment of representational devices, (ii) reflections on and proposal for how to improve representational devices, and (iii) efforts to implement the proposed improvements’ (Cappelen & Plunkett, 2020). As ‘representational devices’, we consider concepts, which we understand roughly as a ‘consistent component of thoughts’ (Cappelen & Plunkett, 2020). The structure of this article is as follows: in the second section we develop the concept of the racialisation of individuals. We also discuss the processes contributing to the rationalisation of the individual that may be medically relevant (using the example of hypertension). We finish with a discussion on the potential application of our proposition to institutional guidelines about the use of racial categories in biomedical research.

## Racialisation and biomedical research

While there are many definitions of racialisation (Barot & Bird, 2001; Gans, 2017), most of them refer to processes by which individuals or groups are interpreted as representatives of a certain race. To present how the concept of racialisation can be applied in biomedical research we propose to redefine it as a continuous process operating through several pathways (and their complex interactions) by which an individual comes to be understood by others (as well as recognising themselves) as a representative of a major biological entity and human lineage formed due to reproductive isolation, in which membership is transmitted through biological descent (cf. Hochman, 2019,9).

We analyse racialisation in the context of individuals because everyone can be racialised differently depending on the level we are considering. In other words, each person may be assigned to a few different ethnoracial categories, not only when it comes to their self-affiliation but also regarding the different levels at which the processes of racialisation take place (these categorisations can also change with time). Establishing which process of the racialisation of individuals is relevant for a particular study is necessary for proper construction of the reference classes and selection of the research participants as well as interpretation of the study results.^10^ However, if the effect of rationalisation of an individual is their recognition as a representative of a specific ethnoracial group, studying the processes of racialisation of the individual also requires knowledge about the racialisation of groups, i.e. the processes that led to the formation of beliefs that there are certain ethnoracial groups, and that all their members share certain psychological and biological features (cf. Krieger, 2012, p. 939).

**Fig. 1:**
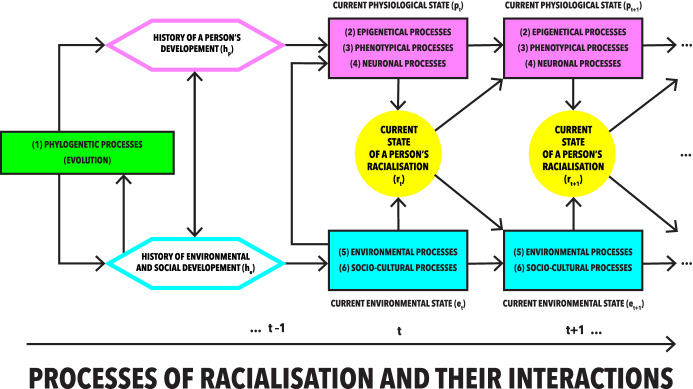
This diagram presents different levels through which processes of racialisation of an individual take place: (1) phylogenetic, (2) epigenetic, (3) phenotypic, (4) neuronal, (5) environmental, (6) socio-cultural. The current state of a person's racialisation at time t + n (yellow circles) is directly co-determined by current physiological states (2–4) and current environmental states (5–6), which were shaped by the history of a person's development as well as the history of environmental and social development. The intensity, importance, and interactions between these processes (as well as the current state of a Person's racialisation) change over time. At the background of this, there is a phylogenetic level (1) at which evolutionary processes occurs

When it comes to the factors affecting and co-shaping racialisation, we distinguish a few different pathways through which these processes take place: (1) phylogenetic, (2) epigenetic, (3) phenotypic, (4) neuronal, (5) environmental, (6) socio-cultural, cf. Figure 1. To describe these different types of processes of racialisation and their relationships, we use the term ‘level’. Thus, we write, for example, about the level of neuronal processes of racialisation or the level of environmental processes of racialisation. However, we do not interpret relations between these levels as strictly separable, stable or hierarchical.

Our choice of six main types of processes of racialisation is supposed to indicate the complex nature of racialisation as well as a variety of health issues that may be analysed with this concept and that are now researched through the prism of the categories of race and ethnicity. It is based on publications on the impact of various biopsychosocial factors affecting so-called racial differences in health (Matsui et al., 2019; Musemwa & Gadegbeku, 2017; Persad-Clem et al., 2022; Rubin, 2016). We have reconstructed and unified the types of factors influencing health described, among others, in the above articles. We have subsequently analysed them in the context of discussions on racialisation (Hochman, 2017, 2019) and general biosocial human development (Baltes et al., 2006). On this basis, we have constructed a model that reflects both the state of knowledge on so-called racial differences in health, and discussions on racialisation, ethnoracial beliefs and racism and their impact on human development.

Our specification of certain levels is intended to structure and distinguish different pathways by which individuals are being racialised (and racialise themselves), and by which racialisation affects their health. Each level can be distinguished by the occurrence of certain mechanisms specific to it (e.g. changes in the genetic pool at the phylogenetic level, changes in gene expression at the epigenetic level, changes in brain functioning at the neuronal level, changes in the structure and functioning of the body at the phenotypic level, changes in the material and symbolic environment at the environmental level, socio-cultural changes at the level dedicated to them). Our model is neither universal nor final. It may be revised or specified to adapt to different problems (e.g. focusing on processes that we have not specifically distinguished, such as institutional, economic, etc.), or owing to the development of science (e.g. our knowledge about racialisation).

As the processes of the racialisation of an individual last through their whole lifetime, we propose to analyse them considering the passage of time, i.e. the state of a person's racialisation at time *t* (at some or all of its levels) may be different from the state of a person's racialisation at time *t* + 1. Their ‘initial’ state is determined by the history of human biological and cultural evolution. The phylogenetic level (1) is the level at which evolutionary processes occur. These processes are usually studied by focusing on the perspective of populations rather than that of individuals. Yet, owing to these processes (co-shaped by environmental and socio-cultural processes) individuals endowed with a unique set of genes (genome) emerge.

The history of a person’s physiological (physical and psychic) development is co-determined by genome whose expression is continuously modulated by epigenetic processes. It is also a reflection of a general phenotypic plasticity, including neuronal processes. All these processes are modulated by a person’s life experiences (the history of a person’s development) taking place in a certain societal and environmental context (a history of environmental and social development). The main question asked when it comes to the analysis of the epigenetic level (2) is how a given genome interacts with a particular environment (including its socio-cultural aspect). The phenotypic level (3) is a broader level, at which all the processes shaping and changing physical state of an individual (phenotypic plasticity) occur, not only in the context of interactions between genome and environment, but also other processes in the body and affecting the body (e.g. the impact of environmental factors on the functioning of specific organs etc.). The neuronal level (4) is the level where analysis of the processes co-shaping human brain structures (neuronal plasticity) occurs. The embodied experiences of the individual (shaped for example through education, by the entire history of human social development) affects their self-construction, behaviour, emotional health and cognitive functioning. The environmental processes (5) are processes that occur in the material and symbolic environment of a person (e.g. climate change, urban development, migration, the development of architectural exclusion). At the level of socio-cultural processes (6), the focus is on the broadly understood socio-cultural factors (the racialisation of groups, political and institutional decisions, local traditions, stereotypes, economic exclusion or privilege etc.) affecting a person, as well as those in which a person actively participates.

All these processes interact with each other and co-shape each other in a complex network of mutual relations. It is crucial to underline that the current state of a person’s racialisation itself is not homogeneous or stable. Rather, it is a manifold realisation of the multilevel processes taking place in their body and environment—a person may be racialised in different ways on each level. The intensity, importance and interactions of these processes (as well as the current state of a person’s racialisation) change over time.

### The processes of racialisation and human health: the case of hypertension

To present our conceptualisation and explain how it can improve medical research and practice, let us look at the case of hypertension in the African diaspora in the US (cf. Kuzawa & Gravlee, 2016). The so-called racial differences in the risk, course and treatment of hypertension and other cardiovascular diseases have been carefully analysed by several researchers (Borrell, 2009; Boyle, 1970; Deere & Ferdinand, 2020). It is said that African Americans living in the US have rates of hypertension about 45% higher than among whites (Mozaffarian et al., 2016; Valderrama et al., 2013). This problem is very often examined through the prism of racial categories understood in two ways: biologically (Cooper & Zhu, 2001; Nanba et al., 2019; Zilbermint et al., 2019) and socio-culturally (Mendes et al., 2018; Williams & Neighbors, 2001). However, it is difficult to grasp unambiguously the causes of the so-called ‘racial differences’ in blood pressure or other cardiovascular conditions and their treatments using the category of race. Aside from the issue of the existence of human races in biological or cultural meanings, hypertension is a complex condition caused by many different biological and socio-cultural factors as well as their interactions (Musemwa & Gadegbeku, 2017). This blurs the definitions of race used in research on this subject even more, leaving room for misinterpretation of the data and research results (Lee, 2009; Sleight, 2000; Sun et al., 2012; Vyas et al., 2020).

In the following subsections we will characterise each of the levels at which the processes of racialisation take place and briefly discuss these in the context of research and hypotheses on so-called ‘racial difference’ in hypertension.

#### Phylogenetic processes

Phylogenetic processes are responsible for the diversity in the genetic pool of *homo sapiens*. When it comes to biomedical research, it is a fact that genes that are more prevalent in populations that correspond with some differences in people’s susceptibility to diseases and reactions to pharmacological therapies. Research on genomic diversity across populations plays a major role in clinical genomics, pharmacogenetics or genetic epidemiology, that is, assessing whether a certain gene variant is more or less common in a particular ancestral group (Kapoor, 2016). In recent years, important links between genetic markers and the risk factors for diseases or responses to treatment (such as metabolism, toxicity and the efficiency of drugs) have been discovered (Motsinger-Reif et al., 2013; Kapoor, 2016).

For the problem we are discussing, this is relevant for three reasons. First, genetic diversity is, to a point, responsible for the differences in people’s appearance. These differences usually provide a naïve justification for the socio-cultural processes of racialisation responsible for the development of folk racial categories. Second, attempts to scientifically divide, categorise and order genetic diversity by means of folk racial categories biologise them and strengthen the belief in the existence of human races. Third, genetic differences between individuals (occurring more often in representatives of certain populations) may be biologically relevant from a medical perspective and need to be studied, but not using the category of race.

The idea that the category of race corresponds to certain clear and distinct genetic differences between humans has been criticised for years (cf. Lewontin, 1972; Sussman, 2014; Sussman et al., 2016). The existence of biological human races understood as racial genetic clusters has been officially questioned, inter alia, by the American Association of Physical Anthropologists (AAPA), which published a statement on this issue [released first in 1996 and revised in 2019 (Fuentes et al., 2019)]:

Race does not provide an accurate representation of human biological variation. It was never accurate in the past, and it remains inaccurate when referencing contemporary human populations. Humans are not divided biologically into distinct continental types or racial genetic clusters. …

Humans share the vast majority (99.9%) of our DNA in common. Individuals nevertheless exhibit substantial genetic and phenotypic variability. Genome/environment interactions, local and regional biological changes through time, and genetic exchange among populations have produced the biological diversity we see in humans today. Notably, variants are not distributed across our species in a manner that maps clearly onto socially recognized racial groups. This is true even for aspects of human variation that we frequently emphasize in discussions of race, such as facial features, skin color and hair type. No group of people is, or ever has been, biologically homogeneous or ‘pure.’ Furthermore, human populations are not—and never have been—biologically discrete, truly isolated, or fixed (Fuentes et al., 2019).

The leading idea of this document is that it is true that there are genetic and phenotypic differences between humans. It is also true that genetic diversity resulting from evolutionary processes has led to the emergence of a huge variety of human phenotypes: a multitude of skin tones, different body structures, hair textures and eye colours. But these differences do not create biologically understood races (which do not exist), but merely provide the basis for the emergence of socio-cultural racial classifications (i.e. folk racial beliefs). Thus, while there are no human races, the phenotypic multiplicity (which is a result of the phylogenetic processes) was pigeonholed into just a few categories (which varied in different cultures) that were labelled as racial (Haslanger, 2008, 2010; Mallon, 2018).

However, folk racial categories based on people’s appearance still operate in science and other dimensions of social life. This leads us to one more way in which genetic diversity contributes to racialization—these folk racial classifications often organise and co-shape scientific practice; for example, they form the basis of the official institutional guidelines for racial classifications in the US. Researchers use them to divide and categorise people according to their genetic differences, and often argue that, even if essentially understood human races do not exist, the category of race refers to some biologically real entity (Rosenberg et al., 2002; Sesardic, 2010; Spencer, 2014). This way of thinking tries to escape accusations of pushing through racist and scientifically unreliable theories about the existence of human biological races while preserving the use of the category of race as grabbing some important genetic differences between populations. Yet, there are many indications that similar argumentations only perpetuate misconceptions about the existence of human races (Hochman, 2013, 2016; Spencer, 2015; Malinowska, 2016; Winsberg, 2022).

There are a growing number of arguments and increasing evidence that racial categories fail to reflect the global distribution of genetic variability (Huddart et al., 2019; Popejoy et al., 2020). Thus more and more researchers stand on the position that its use (e.g. as self-reported race affiliation based on folk beliefs about races) in genetic research on people’s health is a poor proxy for genetic ancestry and may lead to erroneous conclusions (Borrell et al., 2021; Kuzawa & Gravlee, 2016). This is one of the reasons that some researchers in the field of genetics and genomics are moving away from using the category of race in their research and replacing it with the category of ethnicity or ancestry (Byeon et al., 2021).

Populational differences in the frequencies of certain alleles in most cases result from the geographical or cultural isolation of populations (examples of such ‘local’ adaptations are populations with genes related to malaria resistance (Hedrick, 2011), high-altitude adaptation (Simonson et al., 2010) or lactose tolerance (Tishkoff et al., 2007)). Thus, some researchers argue that ‘geography has historically been the greatest predictor of genetic variation between human populations, with genetic distance increasing as geographic distance increases’ (Huddart et al., 2019, p. 1258), and that population grouping should be focused on demo-geographical classification (e.g. as ‘seven geographically-defined groups: American, Central/South Asian, East Asian, European, Near Eastern, Oceanian, and Sub-Saharan African, and two admixed groups: African American/Afro-Caribbean and Latino’ (Huddart et al., 2019, p. 1256; cf. Popejoy et al., 2020)), and not on folk racial classifications as recommended, for example, by US institutions such as the FDA. As they do not overlap with folk racial categories, demo-geographical categorisations could be understood in terms of classes constructed for specific scientific purposes and not as categories reinforcing folk racial beliefs. However, while demo-geographical categories are much more accurate than racial classifications for use in genetics and genomics, they still represent idealisations and generalisations. Further work in refining and clarifying them is also continually needed.

To illustrate the processes of phylogenetic racialisation, let us now return to the matter of hypertension. The ‘racial differences’ in the risk, course and treatment of hypertension were repeatedly explained by genetic differences between populations categorised as races (Cooper & Zhu, 2001; Havlik & Feinleib, 1982; Zilbermint et al., 2019). And although (as mentioned above) such categorisation is increasingly criticised, some researchers are still assured of the genetic origin of the predisposition to hypertension in African Americans (Nanba et al., 2019; Spence & Rayner, 2018). This is mainly owing to the popularity of the highly controversial ‘slavery’ hypertension hypothesis. According to this concept, the predisposition to high blood pressure in representatives of African diasporas is a result of a high mortality from salt-depleting conditions that selected ‘salt-retaining genotypes’ among enslaved Africans (Grim & Wilson, 1993 cf. Kuzawa & Gravlee, 2016; Spence & Rayner, 2018). Although it was repeatedly criticised as unjustified, pseudoscientific and racist (Armelagos, 2005; Kaufman & Hall, 2003; Lujan & DiCarlo, 2018), this theory inspired other researchers to look for unique genetic dispositions in this population. Until now there have been many hypotheses linking hypertension with the frequent occurrence of certain genes among African Americans or Latino people (Adeyemo et al., 2009; Kochunov et al., 2011). However, they remain inconclusive since, for example, the authors could not replicate their results with different cohorts, or the analysed genes were found to be common in both African and non-African groups (Franceschini et al., 2013; Kidambi et al., 2012; Musemwa & Gadegbeku, 2017). It is therefore increasingly unlikely that ‘racial differences’ in hypertension will find an explanation at the level of phylogenetic processes. And even if the variance in the frequency of the occurrence of certain genes in different populations plays an important role here, this is not a ‘racial difference’, but rather a genetic difference, better captured by the application of demo-geographical classifications. Referring to concepts such as the ‘slavery hypothesis’ and fixating on the genetic differences between so-called ‘races’ can only support the myth of the existence of human biological races and thus affect the racialisation of individuals.

#### Epigenetic processes

While so far the analysis of the genetic mechanisms behind the differences in the incidence of hypertension has been rather unsuccessful, there is increasing evidence that some of this may be due to epigenetic processes, including the epigenetic processes of racialisation.

Generally, the functioning of genetic material passed on to subsequent generations can be modulated through epigenetic processes. These take place throughout the life of an organism starting from the earliest stages of its development, and are dependent on biochemical factors, both at the intracellular level and more generally, such as in the endocrine or physiological system (Bradley & Corwyn, 2002; Propper & Rigg, 2007). The regulation of epigenetic processes may depend not only on the experiences of the individual, but also on the experiences of their ancestors: more precisely, in certain situations, to some extent, they may be epigenetically transmitted between generations (Hyde, 2015; McGowan et al., 2009; Meaney, 2010).

Epigenetic processes (particularly DNA methylation) can affect the racialisation of an individual in three ways. First, environmental factors such as the exposure to stress, the family environment, air pollution, poor diet or a lack of sleep alter people’s genetic expression. There is a growing amount of research showing that the socio-cultural, environmental and institutional processes of racialisation (leading to significant social inequalities) might be important drivers of the so-called ‘racial’ disparities in health (Ahmad et al., 2017; Mancilla et al., 2020; Vick & Burris, 2017). The long-term exposition to stress related to racism can disrupt a person’s ‘normal’ physiology and result in chronic diseases. Moreover, Wright et al. (2020) talk about the ‘high effort coping strategies needed to counteract the burdens of racism, which has been associated with higher rates of hypertension and depression in Blacks’. Thus, there is a significant association between perceived racial discrimination and the higher incidence of cardiovascular disease and high blood pressure as well as stroke, chronic kidney disease, depression, schizophrenia, diabetes and certain cancers (Barcelona de Mendoza et al., 2018; Mulligan, 2021; Salas et al., 2020). Therefore for some researchers the level of epigenetic processes might be much better for studying the ‘racial differences’ in developing hypertension and other cardiovascular diseases then the level of genes (Kuzawa & Gravlee, 2016).

Second, epigenetic processes can lead to the embodiment of trauma experiences caused by the racialisation of a person’s ancestors (Conching & Thayer, 2019). For instance, there are studies indicating that the effects of long-term stress or trauma can be passed down the generations through epigenetic mechanisms (Dubois & Guaspare, 2020; Krippner & Barrett, 2019; Lehrner & Yehuda, 2018). Health problems (such as the disturbed regulation of cortisol secretion, preterm birth, low birth weight elevating the risk of hypertension, and a tendency to obesity often leading to cardiovascular diseases) associated with traumatic experiences might be transmitted epigenetically, especially through the maternal line, even if the stress-producing stimulus is no longer present (Dias & Ressler, 2014; Saldaña-Tejeda, 2018). There are studies finding that women who reported ethnic or racial discrimination during pregnancy had worse self-rated health, higher evening cortisol levels and gave birth to infants with smaller birth size and higher cortisol reactivity (affecting their psychological and physiological wellbeing), all independent of ethnicity and material deprivation (Barcelona de Mendoza et al., 2018; Thayer & Kuzawa, 2014, 2015). Such issues caused by the experience of systemic racism and other traumas (migration, experience of war, etc.) inherited from their ancestors significantly reduce the ability of an individual and their social group to improve their socio-economic situation. They also contribute increased levels of stress and trauma in these individuals, aggravating existing health problems and creating new ones. As a result, we are dealing with ‘the matrilineal family cycles of biosocial suffering and exclusion in racialized groups’ (Ramirez-Goicoechea, 2013, p. 80; cf. Conching & Thayer, 2019; Jadotte, 2019).

Finally, health issues resulting from epigenetic processes may also contribute to the development of racial stereotypes. For instance, lower birth weight (more common in the representatives of racialised minorities)^11^ increases the risk of too much body weight in the future. Such a tendency, reinforced by other phenotypic processes and social inequalities, may produce a growing number of racialised individuals struggling with obesity (Duggan et al., 2020; Valdez & Deomampo, 2019; Williams, 2021). The high percentage of obese people in minority communities leads to the development of harmful stereotypes and their further social stigmatisation and racialisation (Dovidio et al., 2018; Machado et al., 2021; Sanders, 2019). The stigma resulting from high body mass can additionally induce hypertension and exacerbate the health problems of racialised individuals (Abel et al., 2021; Blascovich et al., 2001; Levy et al., 2008).

#### Phenotypic processes

Phenotypic processes are all the biological processes in the body related to its development and phenotypic plasticity. Phenotypic plasticity is the ability of a body to adapt to its environment and change under the influence of experience. More precisely, ‘it describes the ability of single genotypes to produce distinct phenotypes in different environments. […] When populations encounter environmental change, plastic traits will result in phenotypic alterations without genetic response’ (Mallard et al., 2020, p. 2429; cf. Pigliucci, 2001). Early life experiences and environments are particularly important in this context, as they can have a profound and persistent impact on traits expressed throughout a person’s lifetime, affecting behaviour, health, disease risk and mortality rates (Lea et al., 2017, p. 162; cf. Leow, 2017). To a large extent, epigenetic mechanisms (modifying gene expression) are responsible for phenotypic plasticity (Leow, 2017; Mallard et al., 2020). However, experiences and environments can shape patterns of health not only through epigenetics, but also through the natural and inevitable processes of body development and ageing, sometimes called ‘wear and tear’ (Kuzawa & Gravlee, 2016; Payson, 2017).

The cumulative experiences of daily life shape human bodies and their immune system. In some cases, they lead to greater fitness and strength, for example, thanks to regular exercising and a healthy diet (Dey et al., 2016; Ghalambor et al., 2007; Kumar et al., 2021). At other times, the cumulative impact of adverse environments can result in health deterioration (Kuzawa & Gravlee, 2016; Vineis et al., 2020). In many cases, phenotypic racialisation is affected by socio-cultural and environmental processes modulating the patterns of a person’s health behaviours, as well as external factors (e.g. conditions in the working environment and place of residence) that affect the condition of their body. However, phenotypic processes can also, in turn, significantly affect how a given individual is racialised and contribute to the formation of the socio-cultural processes of racialisation, as they can form the basis for the emergence and persistence of racial stereotypes. For example, they co-shape muscle and fat structure, which directly influences people’s appearance and behaviour. This makes representatives of socio-culturally racialised groups (e.g. who eat in a similar way, have similar habits, economic status, etc.) often also look similar.

To clarify this issue, let us return for a moment to the problems of hypertension and obesity.^12^ These two conditions are closely linked, not only owing to the impact of stress resulting from the stigma related to high body mass mentioned in the previous subsection. Like hypertension, obesity is more prevalent in the representatives of minorities. In the US (between 2015 and 2016), about 40% of adults had problems with obesity (Hales et al., 2017). Among them, there are significant differences between representatives of groups racialised as Black or Hispanic and others. The prevalence of obesity was lower among non-Hispanic Asian adults (12.7%) than among all other races and Hispanic-origin groups. ‘Hispanic (47.0%) and non-Hispanic Black (46.8%) adults had a higher prevalence of obesity than non-Hispanic white adults (37.9%)’ (Hales et al., 2017). Black (54.8%) and Hispanic (50.6%) women are most at risk of obesity. This is largely owing to differences in the socio-cultural racialisation of these groups and associated social inequalities. There are studies indicating that ‘obesity impacts blood pressure through multiple mechanisms including enhanced sympathetic activation, increased salt sensitivity, activation of RAS, and glomerular hyper-filtration with subsequent renal injury. Obesity–hypertension often exists within a cluster of risk factors (insulin resistance and dyslipidemia) which have additive cardio-vascular risk’ (Musemwa & Gadegbeku, 2017, p. 129; cf. Price et al., 2018; Hall et al., 2019). These problems can be determined largely by epigenetic processes. However, the phenotypic processes of racialisation are not without significance in this context—the greater the racial oppression, stress and workload, etc., the more the body wears out, with worsening health problems, including the risks of obesity and hypertension (Mayin et al., 2021; Santana-Cárdenas, 2020; Tomiyama, 2019).

As for the impact of the above phenotypic processes on racialisation, this is like the impact of epigenetic processes on the development of racial stereotypes. While obesity is a serious disease, people with high body mass are constantly additionally stigmatised as ‘lazy’, ‘sloppy’ and ‘less intelligent’. They also have a worse position in the labour market and often encounter microaggression (Manns-James et al., 2020; McCubbin & Antonio, 2012). Such stereotypes, which frequently apply to whole racialised groups (Dovidio et al., 2018; Machado et al., 2021; Sanders, 2019), deepen social and health disparities.

#### Neuronal processes

It is the neuronal processes, along with other processes taking place in the body (phenotypic and epigenetic processes) and co-shaped by processes altering the person (environmental and social processes), that are co-responsible for people’s psychological construction, co-regulating their cognitive functioning and behaviour as well as co-determining their mental health. Neural processes lead to the embodiment of a person's experiences resulting from neuroplasticity. Neuroplasticity is the ability of nerve cells to adapt, change, regenerate and ‘learn’. The structure of the human brain is shaped to facilitate and strengthen neurocognitive processes specific and/or common to a given socio-cultural environment and repetitive behaviours (Chiao, 2018; Wang & Orchard, 2017; Wexler, 2011). As a result of such processes, the neuronal structurisations of different people’s brains might vary significantly.^13^ Thus, owing to neuroplasticity, each experience co-shapes the human brain, contributing to how the person perceives the world and reacts to it, recognises and interprets stimuli, focuses attention, etc.

Neuronal processes can affect racialisation in a few different ways. First, they contribute to the emergence of fundamental differences in information processing (modulating the patterns of activation of certain groups of neurons, the brain structure, etc.) and the outcome between representatives of different cultural environments. While research on neurodiversity and the impact of socio-cultural factors, etc. on the human brain are extremely important, they often lead to the scientific essentialisation and racialisation of some populations (Martínez-Mateo et al., 2012, 2013). This is because researchers studying these problems often use very general racial and cultural categories as well as universalising their findings to all members of some groups (e.g. claiming that Asians ‘have collectivistic brains’ while Americans have ‘individualistic brains’ (Zhu et al., 2016; cf. Nguyen-Phuong-Mai, 2019) or that there are some relevant differences in neuronal structures between Chinese and Caucasians (Lou et al., 2020; Tang et al., 2018)). Generalising that there are brain structure differences between all representatives of certain races, ethnicities or other social groups (even if told that these differences are caused by cultural factors) is methodologically unreliable, biologises race and strengthens racial stereotypes. These problems arise when researchers are reaching for evolutionary explanations (Malinowska, 2016; Trujillo et al., 2021).

Second, neural processes are correlated with cognitive processes, and therefore also with how people perceive themselves at any time, how they reason, how they perceive reality and how they react to it. They co-construct people’s personal and social identities (changing across their lifetime), collective identifications, social cognition as well as behaviour (Brown, 2020; Haslam et al., 2021; Scheepers & Derks, 2016). Thus, neuronal processes, co-shaped by the history of people’s development (their embodied experiences), are co-responsible for which folk categories of race people identify with and how they understand them. These categorisations are fluid and change with time, owing to factors such as ‘moving in or out of relationships, neighbourhoods, social class groups, or cultural practices, affecting one’s perception by others and one’s sense of oneself’ (Heyes, 2009; cf. Saperstein & Penner, 2012, 2016).

Moreover, there are studies indicating that neuronal processes have a direct impact on human health. For example, stress resulting from the daily experience of racism, xenophobia, social exclusion or trauma related to migration can significantly affect a person’s personal identification and emotional wellness (e.g. leading to the development of PTSD and depression as well as hypertension). Studies suggest that being socially racialised as a member of a minority affects the development of the brain and elevates the risk of first-episode psychosis regardless of a person’s socio-economic status and living conditions (Kirkbride et al., 2017; cf. Malinowska & Żuradzki, 2017, 2020). In order to understand the influence of such factors on a person’s health, one must analyse not only their social experiences, but also how they develop their intersected social identities (among others, how they are racialised by others and how they racialise themselves).

Finally, there are psychological and perceptual mechanisms that influence intergroup behaviours and biases, for example, the extent of empathising with representatives of other social groups and the tendency to ‘dehumanise’ and ‘unify’ them, etc. (Malinowska, 2021; Van Bavel & Cunningham, 2012). These mechanisms contribute significantly to the epistemic unification of the representatives of other groups (their more or less unconscious and automatic perception as homogeneous groups) (Malinowska, 2021, cf. also Malinowska & Żuradzki, 2022) and the socio-cultural racialisation of individuals, for example, owing to their influence on the creation and reinforcement of stereotypes and racism. However, it should not be forgotten that even though the appearance of some of these mechanisms might have certain adaptive purposes (e.g. to improve cognitive processes, enabling quick reactions to representatives of other groups who may be dangerous, etc.) and therefore be the result of phylogenetic processes, they have probably not evolved to regulate human interracial interactions (Kinzler & Spelke, 2011; Kinzler et al., 2007, 2009; Malinowska, 2016).

Although racist or xenophobic views and behaviours are related to specific neuronal processes, it is not the case that all people are doomed to behave that way owing to their biological, embodied origin. Such an interpretation may lead to the biologisation of racism (Kahn, 2012; Majeed, 2021). Actually, it is just the opposite—the socio-cultural processes of racialisation are mainly responsible for the fact that people learn to recognise those with a different skin colour as ‘others’ and apply all sort of racial stereotypes to them (Malinowska, 2016). Thus, their precise analysis and understanding of how far these mechanisms depend on factors such as education, motivation or values shared by people (Lebrecht et al., 2009; Sheng & Han, 2012) are among the main challenges of social psychology and neuroscience. Knowledge from a broader perspective can help in the fight against racism and xenophobia.

As for the influence of the neuronal processes of racialisation on hypertension, in this area there is also a growing body of research showing that they may be somewhat important in this case. First, it is about the relationship between emotional states, stress and high blood pressure. It is here that stimuli coming from the whole body are processed and interpreted, and based on this, specific predispositions to action are generated. Among other things, an individual’s previous socio-cultural experiences affect what social situations they consider stressful and which of them will trigger a feeling of panic. For example, many representatives of minorities, especially Black communities in the US, experience high levels of stress at the sight of police officers, while the same situation might be emotionally neutral for white representatives of the middle class (Pryce et al., 2021; Smith Lee & Robinson, 2019). Moreover, people who experience various forms of social exclusion and exploitation, including racism, are often prone to PTSD, depression and anxiety (Bale & Jovanovic, 2021; Fernando, 2018; Khaylis et al., 2007). This significantly elevates the risk of developing hypertension and obesity (Cuevas et al., 2017; Derks & Scheepers, 2018; Hill & Thayer, 2019).

Finally, owing to the psychological mechanisms responsible for the epistemic unification of representatives of other groups, racialised individuals are exposed to stereotypical (often based on racial stereotypes and folk racial categories) treatment by diagnosticians and other health professionals (Greer, 2010; Moskowitz et al., 2012; Puddifoot, 2019). Implicit bias can affect clinical judgement and decision making (Puddifoot, 2019), as well as influencing the way in which biomedical research is conducted and the conclusions drawn from it (Malinowska, 2021). They can also lead to discriminatory practices such as making assumptions about a patient’s ability to afford services, apathy in reaching diagnoses or the avoidance of touch during physical exams (Greer, 2010). In response, patients experiencing discriminatory behaviours often respond by not keeping appointments with providers perceived as racially discriminatory. Thus, experiencing racial discrimination in clinical encounters may be a significant barrier to appointment attendance for hypertensive African American patients and deepen the existing disparities in healthcare (Greer, 2010).

#### Environmental processes

We understand the environmental processes of racialisation as both influencing the humanecological environment (climate change, pollution, transformation and destruction of green areas) and co-creating and shaping the social environment in which people function (urbanisation, architectural trends and policies, the creation of various borders, ghettos and reservations, etc.).

The origins and course of human biological and cultural evolution depend on the climate in which our ancestors developed. ‘Difficult’ climates as well as their changes have influenced the history of human migrations (Mauelshagen, 2018). The paths of early migration especially are correlated with the emergence of subgroups with unique phenotypic features. For instance, inhabiting different territories was one of the major factors affecting the development of adaptive characteristics, such as the lighter skin pigmentation of residents of areas with less access to the sun with less access to the sun that helps in the synthesis of vitamin D (Jablonski & Chaplin, 2017) or high-altitude adaptation (Simonson et al., 2010). In brief, human migration is co-responsible for the remarkable diversity found in the genetic pool of *homo sapiens* (Curtin, 2018; Manning & Trimmer, 2020; Schlebusch & Jakobsson, 2018).

In fact, people’s migrations caused by environmental processes continue to ‘mix up’ human genes (Raghavan, 2018). For example, the current climate crisis (additionally strengthened by socio-cultural factors, such as armed conflicts or economic crises) is contributing to the increasingly frequent migration of representatives of many populations (Cattaneo et al., 2019; Hugo, 2011; Radel et al., 2018). Thus, while environmental processes are contributing to the phylogenetic emergence of different populations, they also constantly make it harder to use any demo-geographical classifications efficiently in genetics or pharmacogenomics. They also make the realist, biologised and essentialist interpretations of the category of race completely inadequate and useless when applied to humans (Winsberg, 2022).

Migrations contribute to the racialisation of individuals at the levels of socio-cultural and neuronal processes. First, they can increase social tension, leading to racist behaviours in the communities to which migrants flow because of the frequent categorisation of newcomers in urban areas as racial or ethnic others (Benmayor & Skotnes, 2017; Castañeda, 2017; Sajjad, 2018). Second, changing environments (especially migration) affect the self-identification of individuals. For example, a complete change of environment may lead to self-segregation by representatives of some populations and the strengthening of their tendencies to auto-racialisation (and thus the enhancement of the neural processes responsible for self-identification with particular folk racial categories) (cf. Keskinen & Andreassen, 2017). Because in different social environments (owing to the socio-cultural and institutional processes of racialisation) there are differences in racial categorisations, a change of environment may not only strengthen identification with specific folk races, but may also cause significant changes in a person’s auto-identification (Grill, 2018).

Moreover, representatives of groups who are racialised socio-culturally and/or institutionally are additionally racialised environmentally many times (and inversely—inhabiting specific territories may contribute to their socio-cultural and institutional racialisation), for example, when architecture in some districts is designed to exclude these groups from use^14^ or is intended especially for them. The environmental processes of racialisation affect the human social environment, for example, in the racialisation of schools (Saporio & Sohoni, 2006), or the creation of ghettos (Blessett, 2020; Rothstein, 2021; Wilkes, 2003) or reservations (Hooks & Smith, 2004; Rogerson & Rogerson, 2020; Strauss, 2019), or the organisation of museum exhibitions (Redman, 2016). It very often happens that the inhabitants of racialised districts, reservations, etc. have worse access to healthcare, green areas, cultural facilities, schools or sport objects, which can directly affect their health (Christensen et al., 2008; Hales et al., 2018; Sliwa et al., 2016).

Finally, to analyse the so-called ‘racial differences’ in health, sometimes environmental processes may be even more important than the processes for other levels of racialisation. For example, since the lands of Native American tribes have ‘been targeted by the US, government and the large corporations as permanent areas for much of the poisonous industrial by-products of the dominant society’ (Brooks, 1998, p. 106; cf. Meissner, 2021), American Indians who live on these lands have a high likelihood of being exposed to cancer-causing environmental risk factors (Weaver, 2010). However, as about 78%^15^ of American Indians do not live on reservations, analysis of this problem simply using the category of race (understood as institutional affiliation, self-declaration or in biological terms) ‘may not only fail to track environmental hazards to health, it may also obscure the effects of environmental racism’ (Meissner, 2021, p. S2452).

Let us return to the issue of hypertension. A growing body of research shows that certain characteristics of racialised environments contribute to an increased risk of hypertension and other cardiovascular diseases (Claudel et al., 2018; Payne-Sturges et al., 2021; Schell et al., 2020). Neighbourhoods with characteristics such as high stress segregation, low socio-economic status, high crime and other safety concerns, a poorly built environment (e.g. housing quality and urban infrastructure), unequal resource distribution and environmental exposure are often inhabited by representatives of minorities. Such neighbourhood deprivation has been shown to be associated with a multitude of health outcomes including hypertension, higher cardiovascular mortality and obesity (Cubbin et al., 2006; Lee, 2021). Finally, the stress resulting from a change in environment (e.g. migration) also adversely increases the risk of developing high blood pressure.

#### Socio-cultural processes

There is an argument that the first scientific ‘theory of race’ was introduced by Kant,^16^ who gave it a definition and ‘distinguished it from other concepts such as “species” and “variety”’ (Hochman, 2019, p. 9; cf. Sheidt, 1950; Bernasconi, 2002; Sloan, 2014). However, the word ‘race’ (interpreted as a group of people with a common occupation) was introduced into English much earlier—about the year 1500. The history of the concept of human races was influenced by the history of mankind, its conquests, wars, political and economic goals, scientific achievements, etc. (Montagu, 1962; Mead 1968; Levi-Strauss, 1996; Bernasconi, 2020). Scholars largely agree on the socio-cultural origins of the concept of race (Appiah, 1996; Haslanger, 2008, 2010; Mallon, 2006, 2018; Zack, 2016, 2018). Moreover, such a perspective is popular not only among philosophers and other theorists but also among anthropologists and population biologists. For example, in the AAPA statement we read:

While human racial groups are not biological categories, ‘race’ as a social reality – as a way of structuring societies and experiencing the world – is very real. The racial groups we recognise in the West have been socially, politically, and legally constructed over the last five centuries. They developed in tandem with European colonial expansion and the emergence of American and European societies with well-documented histories of being shaped and structured by racial hierarchies, power inequities, economic exploitation, dispossession, displacement, genocide, and institutional racism. These practices are rooted in assumptions of innate, natural differences between Europeans and other peoples, and systems of racial classification are intimately tied to histories of European settler colonialism, empire, and slavery. Classifying human beings into different races has never been wholly innocent, unbiased, or apolitical; racial classification has long served to justify exploitation, oppression, discrimination, and structural racism. Notably, racial categories have changed over time, reflecting the ways that societies alter their social, political and historical make-ups, access to resources, and practices of oppression. (Fuentes et al., 2019)

This quote highlights the most important features of the concept of race: it is a social, political and legal construction referring to superficial differences in human appearance. However, we agree with Hochman, who argues that while the concept of race has a great impact on human life, it has no real reference. Thus, using the category of race as if it referred to some existing entity with ‘little conceptual value, and so attempting to construct a social metaphysics of race, is a wrong-headed project’ (Hochman, 2017, p. 71). Rather than social races, racialised individuals and groups exist. And just like the biologically interpreted category of race, the social constructivist concepts of race have a direct impact on human life. Ultimately, both approaches are a result of ongoing racialisation processes and significantly affect how these processes are proceeding.

Socio-cultural experiences determine which folk racial affiliation is declared by individuals themselves and how they are categorised by others. These categorisations depend on a huge number of factors, for example, how ethnic diversity functions in each community, the history of the community and its contacts with other populations, the power relations between different social groups in the community, the ideologies and social ontologies that are popular among its members, etc. (Ludwig, 2019). As noted by Audrey Smedley (2011), the worldview shaped by the category of race becomes ‘a culturally structured, systematic way of looking at, perceiving, and interpreting reality. But a fundamental lesson of anthropology is that each society develops its *own* culturally structured way of perceiving and interpreting reality.’

Socially recognised racial classifications are adapted, revised and imposed by certain institutional decisions. Depending on the interests of individual institutions, social groups are divided into races and ethnicities in various ways. These divisions differ not only in the number of distinguished racial and ethnic groups, but also in the way they are identified. Often the way in which institutions categorise populations into race and ethnicity has served to perpetuate relationships of power and exploitation. For instance, there are essential differences in how the official political status of being an African American (AA) and a Native American/American Indian (NA/AI) in the US was determined in the twentieth century. Everyone with any known AA lineage was, owing to a lingering US custom, typically labelled as Black (following the ‘one-drop rule’). In the case of NA/AI, it was (and still is) exactly the opposite—most federally recognised American Indian tribes require the verification of some form of ‘Indian blood quantum’ for official membership (Meissner, 2021). This means that in order to obtain the political status of a NA/AI representative, one must be able to prove a greater or lesser degree of kinship with other official tribe members (although not in relation to actual biological ‘blood relations’ or genetics, but rather a broader social understanding of affinity) (Meissner, 2021; cf. TallBear, 2008):

Through the one-drop rule, blackness in settler colonial contexts is *expansive*, ensuring that a slave/criminal status will be *inherited* by an expanding number of ‘black’ descendants. Yet, Indigenous peoples have been racialized in a profoundly different way. Native Americanness is *subtractive*: Native Americans are constructed to become fewer in number and *less* Native, but never exactly white, over time. (Tuck & Yang, 2012, p. 12)

It is also worth adding here that racial and ethnic political statuses rarely coincide with the racial self-identification of individual people and the conditions/environments in which these people function (Meissner, 2021).

Yet another dimension of socio-cultural racialisation is the fact that being a representative of a racialised minority often translates into the social function, profession and economic status of an individual. People identified as representatives of certain racialised groups have worse access to education (especially higher education) and it is more difficult for them to build professional careers (Byrne et al., 2020; McCarthy, 1990; Wei et al., 2018). All of this, together with the experience of racism, limited access to healthcare and the general experience of social oppression and exploitation can have a significant on a person’s state of racialisation and affects their health (Clark et al., 2014; Mendes et al., 2018; Smedley, 2012).

Finally, the capitalist system enhances socio-cultural processes or racialisation, not only by exploiting certain racialised groups, but also by affirming the so-called racial differences between them. It simply pays to create products for particular ‘races’, thus increasing the demand for them and, at the same time, strengthening racial stereotypes. Racially targeted advertising and selling strategies are analogous to gender marketing (Moss, 2017) and are sometimes called race marketing (Crockett, 2008; Kennedy, 2000; Saha, 2015; Sallaz, 2010).

So how do the socio-cultural processes of racialisation affect ‘racial differences’ in hypertension? Let us start with race marketing. A great example of this practice was BiDiL. which was brought about by the interaction of economic goals, folk beliefs about races and flawed methodology based on racial categories. BiDiL is a drug for a heart failure (it lowered blood pressure, among other effects), which was approved by the FDA in 2005 exclusively for AAs. While its appearance on the market was supposed to prove the great success of pharmacogenomic and personalised medicine, it aroused enormous controversy and criticism (Johnson, 2019; Kahn, 2012; Sankar & Khan, 2005). For one thing, this was because BiDiL originated in the 1980s, when it was intended as a therapy for everyone. Then, owing to research budget constraints and institutional requirements, it was approved ‘based primarily on the data produced by a race-specific clinical trial that enrolled only African Americans. With no comparison population enrolled, there is no basis to make any claims that the drug worked differently or better in black than in anyone else’ (Kahn, 2012, p. 7). However, while there was insufficient evidence of any ‘racial differences’ in response to BiDiL when used for hypertension and other cardiovascular diseases, its introduction to the market significantly strengthened the folk beliefs that there are some biological and especially genetic differences between representatives of different races, and thus the tendency to biologise and medicalise race (Kahn, 2012; Pollock, 2012). Additionally, its production as a race-specific drug deepened social and health inequalities and the racialisation of AAs, as BiDiL costs three times more than any other drug in its class (Johnson, 2019, p. 4).

In the end, it is worth adding that the social processes of racialisation also affect people’s lifestyles, directly translating into the risk of developing hypertension (Kokubo et al., 2019). On the one hand, these are the traditions common to specific groups (e.g. particular diets or the frequency of alcohol consumption) (Musemwa & Gadegbeku, 2017). On the other hand, they include the socio-economic conditions in which people live, the work they perform, their opportunities to spend time on physical activity, etc. Finally, the socio-cultural processes of racialisation (as well as their interactions with those on other levels, e.g. neuronal, epigenetic and phenotypic processes) are the direct causes of stress related to social exclusion, exploitation and the experience of racism, all related to the occurrence of hypertension (Derks & Scheepers, 2018; Kuzawa & Gravlee, 2016; Payne-Sturges et al., 2021). When health problems occur, the method of their diagnosis and treatment also depends to some extent on the racialisation of individuals; for example, on the limited access to healthcare for representatives of minorities and inequities in hypertension control (Bress et al., 2021; Rivara et al., 2022).

### ‘Racial differences’ in hypertension: what does it mean?

Putting all the factors affecting the risk of hypertension in racialised individuals into one bag labelled as ‘racial differences’ tells us very little about the analysed phenomena. While the phylogenetic processes of racialisation are less relevant in this context, all the processes of racialisation taking place on other levels may have a significant impact on its occurrence, course and response to treatment. It is not enough to list them; it is also necessary to analyse their relations and mutual influences in detail. Such knowledge is also important when choosing the right reference class/population for research—there should be different selection criteria for analysing the processes of racialisation at different levels. For phylogenetic processes this might involve, for example, having a specific gene or belonging to a particular demo-geographic; for phenotypic processes, occupation and diet, etc.; for epigenetic processes, being a descendant of representatives of a given community with a specific history; for environmental processes, living in a specific neighbourhood; for socio-cultural and neuronal processes, a sense of belonging to a given social group or cultural education. However, a thorough proposition of such criteria for constructing reference classes for analysing the effects of all the processes of racialisation on health requires constant adaptation to a specific research problem.

Identifying the levels of impact of the processes of racialisation on a person’s health as well as the factors that affect them not only allows the complexity of the phenomenon to be understood. It can help with evaluating what aspects of racialisation are indeed medically relevant to the research subject. We believe that such clarification should also be considered when it comes to institutional guidelines for researchers. Their current shape, which often confuses the cultural and biological concepts of race (such as FDA, 2016, p. 7), does not solve the problem and may still contribute to exacerbating inequality.

## Conclusion

At the very beginning of our article, we pointed out the disadvantages of using the category of race in biomedical research. Therefore, after examining the debates about whether human races exist and how the category of race is used in medicine, we attempted to develop our own concept of racialisation. In Sect. 2, we presented an extensive concept of how the processes of the racialisation of an individual proceed at different levels. We also presented examples of how these processes can affect human health. Our arguments indicate that whenever researchers investigate mechanisms including the processes of racialisation affecting human health, it would be informative if they determine precisely the level of racialisation to which they refer. In other words, it would be less confusing if researchers define precisely which level of racialisation they consider to be biologically significant for the problem under study, rather than just referring to the category of race.

In conclusion, we would like to return to the issue of institutional guidelines (including the guidelines of scientific journals) for the use of racial categories in biomedical research. We believe that our proposal may, at least partially, help to make these guidelines clearer and more comprehensible. We will try to paraphrase some of the passages from the FDA (2016) and JAMA (2021) recommendations using our theoretical framework.

As a first example, we will use a quote from Huang and Temple (2008) found on page 7 of the FDA’s document:

Differences in response to medical products have already been observed in racially and ethnically distinct subgroups of the US population. These differences may be attributable to intrinsic factors (e.g., genetics, metabolism, elimination), extrinsic factors (e.g., diet, environmental exposure, sociocultural issues), or interactions between these factors. (Huang & Temple, 2008)

This statement is very enigmatic and has little explanatory power about how race and ethnicity affect health. Applying our analysis to it could change this statement in the following way:

Differences in response to medical products have already been observed in the representatives of racialised groups on many levels. These differences may be attributable to intrinsic factors (e.g., phylogenetic as well as the epigenetic, phenotypical and neuronal processes of racialisation), extrinsic factors (e.g., the environmental and socio-cultural processes of racialisation), or interactions between these factors.

This small change, introduced in the broader context of the concept of racialisation, makes the quote more informative and creates no unnecessary confusion about what race is, whether human races exist, etc. Thus, it does not just replace set of words with another—it changes the general conceptualisation of racial categories. Similarly, let us return to the guidelines from JAMA:

Identifying the race or ethnicity of a person or group of participants may provide information about the generalizability of the results of a study. However, many people may identify with more than one race/ethnicity; therefore, categories should not be considered absolute or viewed in isolation. (Flanagin et al., 2021a; cf. Flanagin et al., 2021b)

Applying our concept, this could look, for example, like this:

Identifying the state of a person’s racialisation on different levels may provide information about the generalisability of the results of a study and may identify important disparities and inequities. However, this state might vary on different levels and change with time, therefore, it should not be considered absolute or viewed in isolation. Also, one person may simultaneously belong to different reference classes when it comes to racialisation, depending on the level being considered and the time being studied. In order to understand the influence of each level’s processes of racialisation on a person’s health, their intersected and developing social identities should be taken into account.

And the further paragraph:

Aggregate, deidentified demographic information (e.g., age, sex, race/ethnicity, and socioeconomic indicators) should be reported for research reports along with all prespecified outcomes. (Flanagin et al., 2021a; cf. Flanagin et al., 2021b)

could be revised as below:

Aggregate, deidentified demographic information (e.g., age, sex, demo-geographic category, socioeconomic and disadvantage indices) should be reported for research reports along with all prespecified outcomes. The use of self-reported ethnoracial data (e.g., to investigate the impact of racism on health) must be supplemented with the use of other inequality indicators, and justified. The self-reported race and ethnicity are strictly socio-cultural characteristics that might be related to only certain processes of racialisation: primarily neuronal, institutional, socio-cultural or environmental, and even between these levels there may be significant differences between the way a person is classified at the moment (e.g., their sense of belonging to folk categories and their official racial or ethnic affiliation according to institutional categories) and how it affects their health. Identifying ethnoracial identity with disadvantage indices may lead to erroneous results based on ethnoracial stereotypes. Demo-geographic class, like ancestry, is a characteristic more relevant to genomic and some epigenomic analysis and should not be equated with a person’s self-reported race and ethnicity. If the impact of the processes of racialisation is analysed in the study and reported in the results, it should be precisely stated which factors (from which levels of racialisation), as well as which interactions between them, are being referred to.

Again, the introduction of a multi-level and processual conception of racialisation introduces some order to the consideration of how its different aspects affect human health. This is just another example of how it could be used in the future. However, a more detailed methodology for such research needs to be developed. Perhaps it may be based to some extent on *Mechanism Discovery Approach* introduced by Kalewold (2020), but this matter requires further analysis.

We believe that our proposal can be explanatorily useful as it corresponds well with the current state of scientific knowledge. When it comes to biomedical research, it provides an understanding of how various factors influence the health of racialised individuals without the unnecessary confusion resulting from using the category of race. We therefore call for the gradual abandonment of the category of race in favour of the concept of racialisation. We hope that our proposition may allow scientists and practitioners to analyse the impact of racialisation on human health more reliably and prevent further reification and medication of the folk racial categories.

## Footnotes

1 In many countries, racial categories are collected and used in biomedical research and healthcare in a way that depends on their cultural norms (see Sect. 2.1.6). This also affects the terminology: for example, in the UK the term ‘ethnicity’ is usually used and understood as a proxy for ancestral lines. However, the US regulations may influence research outside the US (e.g. if someone seeks FDA approval for use in an American market).

2 NIH Policy and Guidelines on the Inclusion of Women and Minorities as Subjects in Clinical Research (October 2001), available at https://grants.nih.gov/grants/guide/notice-files/NOT-OD-02-001.html.

3 The Office of Management and Budget (OMB) Directive No. 15: Revisions to the Standards for the Classification of Federal Data on Race and Ethnicity (30 October 1997), available at https://obamawhitehouse.archives.gov/omb/fedreg_race-ethnicity

4 Food and Drug Administration Guidance for Industry and Food and Drug Administration Staff on *Collection of Race and Ethnicity Data in Clinical Trials* (2016), available at https://www.fda.gov/regulatory-information/search-fda-guidance-documents/collection-race-and-ethnicity-data-clinical-trials.

5 For fluency in the text, we are using the terms ‘category of race’, ‘racial categories’ and ‘ethnoracial categories’ interchangeably.

6 In the FDA’s document, the geographical criteria for belonging to a particular race (e.g. continent or country of origin) are mixed with institutional criteria (e.g. tribal affiliation) without any proper explanation, for example, ‘American Indian or Alaska Native: A person with origins in any of the original peoples of North and South America (including Central America), and who maintains tribal affiliation or community attachment’.

7 NIH Policy and Guidelines on the Inclusion of Women and Minorities as Subjects in Clinical Research (October 2001), available at https://grants.nih.gov/grants/guide/notice-files/NOT-OD-02-001.html.

8 Examples of research articles from the last few years with titles containing the phrase ‘racial differences’: ‘Genetic and Racial Differences in the Vitamin D Endocrine System’ (Bouillon, 2017); ‘Racial Differences in the Incidence of Cardiovascular Risk Factors in Older Black and White Adults’ (Howard et al., 2017); ‘Racial differences in endometrial cancer molecular portraits in The Cancer Genome Atlas’ (Guttery et al., 2018); ‘Experienced discrimination and racial differences in leukocyte gene T expression’ (Thames et al., 2019); ‘Racial differences in treatment and outcomes in multiple myeloma: a multiple myeloma research foundation analysis’ (Derman et al., 2020); ‘Factors Associated with Racial Differences in Deaths among Nursing Home Residents with COVID-19 Infection in the US’ (Gorges and Konetzka 2021); Racial Differences in a Detroit, MI, ICU Population of Coronavirus Disease 2019 Patients’ (Lazar et al., 2021); ‘Risk Factors for Poststroke Cognitive Decline (Reasons for Geographic and Racial Differences in Stroke)’ (Levine et al., 2021).

9 Adam Hochman defines racialisation as ‘the process or processes through which a group comes to be understood to be a major biological entity and human lineage, formed owing to reproductive isolation, in which membership is transmitted through biological descent’ (Hochman 2019, p. 1245).

10 Nancy Krieger deals with this problem from a different perspective. Krieger’s ecosocial theory of disease distribution ‘conceptualizes the relationship between population distributions of health, levels, pathways, and power to clarify how health inequities constitute biological expressions of injustice’ (Krieger, 2019, 4.10). She proposed a model of embodiment of racial inequality and its implications for health inequalities (2012, p. 937, see also 2012, p. 938; cf. 2005, 2021), which aims to reconstruct how people ‘literally biologically embody exposures arising from our societal and ecological context, thereby producing population rates and distributions of health’ (2012, p. 936).

11 There is also some additional evidence that these differences are not due to genetics, but rather to the environment, socio-economic status and other factors associated with socio-cultural, institutional and environmental racialisation. One of these is the fact that upon arrival, US migrants from the Caribbean and Africa were found to have birth weights statistically almost identical to those of the white ancestors of Europeans. However, with time, the birth weight of their offspring born in the US started to shift towards the lower average African American birth weight (Kuzawa & Gravlee, 2016, pp. 100–101; cf. David & Collins, 1997; Collins et al., 2002).

12 Strength and muscle mass are also often the subject of racial stereotyping, for example in sports (Hextrum, 2020; Sheppard, 2020; St Louis, 2003, 2004).

13 For example, it is known that some blind people can ‘use’ hearing in a similar way to vision (echolocation), and that the brain structures usually attributed to vision may be activated precisely to ‘see’ through hearing (e.g. when they ‘see’ faces ‘with ears’, then the left fusiform ‘face’ area activates) (Chebat et al., 2018; Norman & Thaler, 2019; Tonelli et al., 2020).

14 An example of such practices is building bridges between ‘white’ and ‘black’ districts that are so low that buses could not pass under them in order to prevent people of colour from accessing a public beach in the US (Schindler, 2015).

15 These are data based on self-declaration, and thus illustrate primarily the socio-cultural processes of racialisation.

16 As noted by Hochman: ‘Kant believed that humans contained “racial seeds” (*keime*) that were triggered by the climate, just as a cold climate triggers growth of a second layer of feathers in some birds. Furthermore, one might argue that Kant’s theory was immediately refuted. Kant relied on skin colour to classify the “races”. In 1775, the year Kant published his first essay on “race”, John Hunter showed that people of different so-called races could have the same skin colour, and Georg Forster made criticisms along similar lines (see Bernasconi, 2001, p. 17). “It is ironic”, writes Bernasconi, “that at the very time that Kant was giving the concept of race intellectual coherence, his criterion for distinguishing the different races was collapsing” (2001, p. 17).’ (Hochman, 2019, p. 11).
